# Predictors of poor outcome in out-of-hospital cardiac arrest

**DOI:** 10.1186/cc13679

**Published:** 2014-03-17

**Authors:** JE Petrie, S Brett, R Stümpfle

**Affiliations:** 1 Hammersmith Hospital, London, UK

## Introduction

Out-of-hospital cardiac arrest (OOHCA) causes 60,000 UK and 300,000 US deaths each year. Survival to hospital discharge in the developed world has historically been 7 to 10% with obvious cognitive impairment in 10% of survivors. Primary percutaneous coronary intervention (PPCI) and targeted temperature management (TTM) (or at least hyperthermia avoidance) have been shown to improve survival in comatose patients post OOHCA. There is no reliable method to predict poor outcome on presentation. We aimed to identify factors associated with poor outcome in our single-centre regional referral OOHCA population.

## Methods

We performed a pragmatic single-centre retrospective review over 18 months commencing 1 January 2011 of all patients admitted to our regional OOHCA centre ICU following successful resuscitation from OOHCA. In keeping with guidelines, all patients were assessed for suitability for PPCI and TTM. A good outcome was defined by a Pittsburgh Cognitive Performance Category (CPC) score of 1 to 2 (independence, mild impairment) on hospital discharge. A poor outcome was defined as death or CPC 3 to 4 (moderate to severe impairment, coma) on hospital discharge. CPC scoring was determined from physiotherapy and occupational therapy assessments in the clinical notes. Continuous data were analysed using a two-tailed *t *test or Mann-Whitney *U *test, categorical data with a chi-squared test.

## Results

Sixty-nine patients were admitted to our ICU following successful resuscitation from OOHCA in the 18-month period. Presenting features and outcomes are shown in Table 1.

**Table 1:** Presenting features and outcome at hospital discharge of ICU OOHCA patients

	Good outcome (CPC 1 to 2)	Poor outcome (dead or CPC 3 to 4)	Total	*P ***value**
Age	63.3 ± 16.8	63.6 ± 14.4	63.5 ± 14.7	NS
Male sex	21/26 (81%)	29/43 (67%)	50/69 (72%)	NS
Arrest occurring 08:00 to 20:00	14/19 (74%)	16/36 (44%)	30/55 (55%)	<0.05
VF/VT as initial rhythm	25/26 (96%;	25/43 (58%)	50/69 (72%)	<0.001
Bystander CPR	12/26 (46%)	24/43 (56%)	36/69 (52%)	NS
Time from collapse to ROSC (minutes)	18.3 ± 11	32.3 ± 26	27.1 ± 23	<0.05
Advanced airway management	14/24 (58%)	38/42 (90%)	52/66 (79%)	<0.005
Cardiac cause	24/26 (92%)	30/43 (70%)	54/69 (78%)	<0.05
Angiography	19/26 (73%)	23/43 (53%)	42/69 (61%)	NS
Therapeutic hypothermia	24/26 (92%)	41/43 (95%)	64/69 (93%)	NS
Presenting pH	7.21 ± 0.11	7.08 ± 0.18	7.13 ± 0.17	<0.01
Presenting base deficit (mmol/l)	-7.9 ± 6.2	-13.8 ± 5.9	-11.5 ± 6.7	<0.001
Presenting lactate (mmol/l)	4.3 ± 3.0	8.3 ± 4.5	6.8 ± 4.4	<0.0005

## Conclusion

Good outcome was associated with a shockable rhythm on presentation, cardiac cause, arrest occurring in daylight hours (08:00 to 20:00) and shorter time between collapse and ROSC. Poor outcome was associated with placement of an advanced airway device, lower pH, higher base deficit and higher lactate.

